# Study on the Coupled Relationship Between Dry Density and Mechanical Properties of Geopolymer EPS Concrete

**DOI:** 10.3390/ma19132712

**Published:** 2026-06-24

**Authors:** Juan Gao, Sheng Ye, Ji Yuan, Xiaohong Jian, Haijie He, Yuhao Shang

**Affiliations:** 1 Hangzhou Polytechnic, Hangzhou 311402, China; summer06gg@163.com; 2 Zhejiang Hongxing Construction Co., Ltd., Hangzhou 311500, China; 3 School of Civil Engineering and Architecture, Taizhou University, Taizhou 318000, China; he_haijie@zju.edu.cn (H.H.);; 4 Zhejiang Reborn Kete Testing Co., Ltd., Hangzhou 311122, China

**Keywords:** geopolymer EPS concrete, EPS content, dry density, mechanical properties, compressive strength, elastic modulus, empirical model

## Abstract

Geopolymer EPS concrete (GEPSC) is a promising low-carbon lightweight material for building envelope and thermal insulation applications. In order to investigate the effects of expanded polystyrene (EPS) content on the lightweight characteristics and mechanical properties of geopolymer EPS concrete (GEPSC), specimens with EPS volume contents of 30%, 35%, 40%, 45%, 50%, and 55% were prepared. Dry density, cube compressive strength, axial compressive strength, splitting tensile strength, flexural strength, and elastic modulus were tested, and empirical relationships among the main mechanical parameters were established. The results show that dry density, cube compressive strength, axial compressive strength, splitting tensile strength, and elastic modulus decrease with increasing EPS content, indicating a clear lightweighting–strength reduction effect. The low strength and low stiffness of EPS particles weaken the continuity and load-bearing skeleton of the geopolymer matrix, while promoting more dispersed crack propagation and a more gradual failure process. The correlation coefficients of the proposed empirical models are all greater than 0.90. Lightweighting efficiency analysis indicates that an EPS content of 40–45% provides a favorable balance among weight reduction, strength retention, and stiffness retention. Compared with EPS concrete, GEPSC exhibited 23.5–49.5% higher strength at the same density grade, indicating its good strength retention capacity and potential engineering applicability. These findings support mix optimization, mechanical parameter selection, and engineering application of low-carbon lightweight envelope materials.

## 1. Introduction

With the increasing demand for low-carbon and green development in the construction industry, the sustainability of ordinary Portland cement-based materials has attracted extensive attention because of their high energy consumption and large carbon emissions during production [1,2,3,4,5,6]. Geopolymer materials are inorganic polymeric binders formed through the alkali activation of aluminosilicate precursors such as fly ash and ground granulated blast-furnace slag. Owing to their low-carbon characteristics, potential for solid waste utilization, and favorable mechanical properties, geopolymers are regarded as an important alternative to conventional cementitious materials [7,8,9]. Therefore, the development of lightweight geopolymer composites is of practical significance for reducing both material-related carbon emissions and the self-weight of building components.

Expanded polystyrene (EPS) concrete is a typical lightweight concrete prepared by incorporating EPS foam particles as lightweight polymer fillers or lightweight aggregates. Owing to its low density, good thermal insulation performance, and convenient construction characteristics, EPS concrete has been widely used in wall panels, sandwich panels, thermal insulation layers, non-load-bearing envelope structures, and other lightweight building components [10,11,12,13,14,15].

Previous studies on cement-based EPS concrete have shown that EPS particles can effectively reduce density and thermal conductivity, thereby improving the lightweight and thermal insulation performance of concrete [16,17,18,19,20,21]. At the same time, the mechanical properties of EPS concrete are strongly affected by the characteristics and content of EPS particles. For example, Babu et al. investigated the properties of EPS aggregate concrete containing fly ash, and Miled et al. analyzed the influence of EPS particle size on compressive strength [16,17]. Bouvard et al. further revealed the relationship between the microstructure of EPS lightweight concrete and its mechanical and thermal properties through characterization and simulation [18]. These studies demonstrate that EPS particles are effective lightweight fillers for cement-based composites, while also indicating that their influence on mechanical performance should be carefully considered.

However, EPS particles have low strength, low elastic modulus, and hydrophobic surfaces, and their interfacial bonding with the cementitious matrix is usually weak. As a result, increasing EPS content generally causes significant reductions in compressive strength, tensile strength, flexural strength, elastic modulus, and durability [17,18,19,20,21]. To overcome these limitations, researchers have attempted to improve EPS concrete through fly ash or silica fume incorporation, EPS particle-size optimization, surface modification, fiber reinforcement, and multi-objective mix optimization [13,14,16,17]. Although these studies have provided an important basis for understanding the lightweighting–strength trade-off of EPS-based materials, most of them have focused on ordinary Portland cement systems. The material composition, reaction mechanism, matrix structure, and interfacial characteristics of geopolymer systems are different from those of cement-based systems; therefore, conclusions obtained from cement-based EPS concrete cannot be directly applied to geopolymer EPS concrete.

In recent years, lightweight geopolymer composites have attracted increasing attention because they combine the low-carbon advantages of alkali-activated binders with the functional benefits of lightweight aggregates or fillers. Lightweight geopolymer materials have been developed by incorporating expanded perlite, recycled aggregates, rubber particles, foaming agents, EPS particles, and other lightweight phases [22,23,24,25,26,27]. Kioupis et al. investigated lightweight fly ash-based geopolymer composites incorporating EPS and expanded perlite and reported their physical, mechanical, and durability-related properties [22]. Li et al. developed lightweight ambient-cured EPS geopolymer composites and studied their mechanical behavior [23]. Bian et al. prepared geopolymer-EPS core–shell lightweight aggregates to improve the compatibility between EPS particles and geopolymer matrices [24]. Other recent studies have also explored the mechanical properties, durability, freeze–thaw resistance, and carbon-emission reduction potential of geopolymer EPS concrete [25,26,27]. These studies demonstrate that EPS-modified geopolymer composites have potential for low-carbon lightweight building applications.

In addition to lightweight fillers, fiber reinforcement has also been widely investigated as an effective method for improving the brittleness, crack resistance, tensile performance, and toughness of geopolymer composites. Synthetic fibers, steel fibers, basalt fibers, glass fibers, carbon fibers, and plant fibers have been used to enhance the post-cracking behavior and mechanical performance of geopolymer materials [28,29,30,31]. Ranjbar and Zhang reviewed fiber-reinforced geopolymer composites and pointed out that fibers can improve crack-bridging capacity, ductility, and toughness [28]. Camargo et al. reviewed natural fiber-reinforced geopolymer and cement-based composites, emphasizing the importance of the fiber–matrix interface and fiber durability [29]. Recent studies on plant fiber-reinforced geopolymer concrete further show that renewable fibers can contribute to the development of more sustainable geopolymer composites [31]. These studies indicate that the performance of geopolymer composites is closely related to the interaction among the geopolymer matrix, lightweight phases, fibers, and interfacial transition zones.

Although considerable progress has been made in cement-based EPS concrete, lightweight geopolymer composites, and fiber-reinforced geopolymer materials, studies on geopolymer EPS concrete remain relatively limited. In particular, the systematic influence of EPS particle volume fraction on dry density, compressive behavior, tensile behavior, flexural behavior, and stiffness evolution has not been sufficiently clarified. Moreover, empirical relationships among the key mechanical parameters of geopolymer EPS concrete, such as dry density, cube compressive strength, axial compressive strength, and elastic modulus, are still lacking. Such relationships are important for mechanical parameter prediction, mix design optimization, and engineering application.

Accordingly, in this study, fly ash and ground granulated blast-furnace slag were used as the main aluminosilicate precursors, and an alkali-activating system composed of sodium hydroxide solution and sodium silicate solution was adopted to prepare geopolymer EPS concrete specimens with different EPS particle volume fractions. Dry density, cube compressive strength, axial compressive strength, splitting tensile strength, flexural strength, and elastic modulus were tested to analyze the effects of EPS content on the physical and mechanical properties of geopolymer EPS concrete. Empirical relationships between dry density and strength, cube compressive strength and axial compressive strength, and cube compressive strength and elastic modulus were further established. The results provide experimental evidence and theoretical references for the mix optimization, mechanical parameter selection, and engineering application of geopolymer EPS concrete in lightweight wall panels, thermal insulation components, and low-carbon building materials.

## 2. Materials and Methods

**Figure 1:**
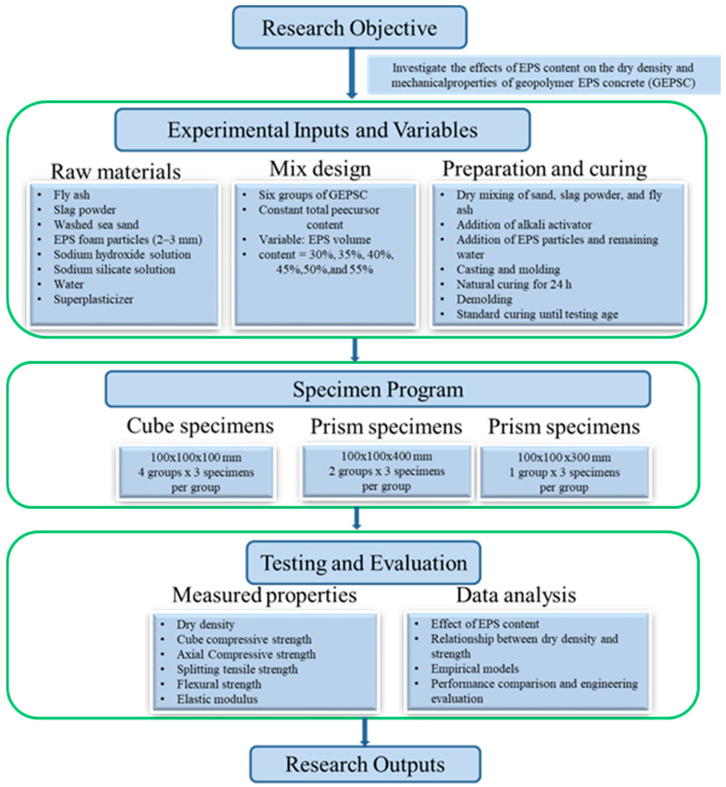
Experimental research scheme.

The overall experimental research scheme is shown in Figure 1. The experimental program was organized according to the sequence of research objective, experimental inputs and variables, specimen program, testing and evaluation, and research outputs.

### 2.1. Raw Materials

The water glass used in this study was supplied by Henan Runzhu Casting Materials Co., Ltd. (Zhengzhou, China); its chemical composition and physical parameters are listed in Table 1. Industrial-grade liquid sodium hydroxide with a mass fraction of 50% was supplied by Shandong Feisheng Chemical Co., Ltd. (Dezhou, China). Washed sea sand was used as the fine aggregate, with an apparent density of 2630 kg/m^3^ and a fineness modulus of 2.64. The binders included Grade II fly ash and S95-grade ground granulated blast-furnace slag; the densities of the fly ash and slag powder were 2.09 g/cm^3^ and 2.89 g/cm^3^, respectively, and their main chemical compositions are shown in Table 2. A polycarboxylate superplasticizer with a solid content of 30% was used, and the EPS foam particles had a particle size of 2–3 mm.

### 2.2. Mix Design

A total of six groups of geopolymer EPS concrete (GEPSC) specimens were designed. With the total binder content kept constant, the EPS particle volume contents were set at 30%, 35%, 40%, 45%, 50%, and 55% to investigate the influence of EPS content on the mechanical properties. The detailed mix proportions are listed in Table 3.

### 2.3. Specimen Preparation and Curing

**Figure 2:**
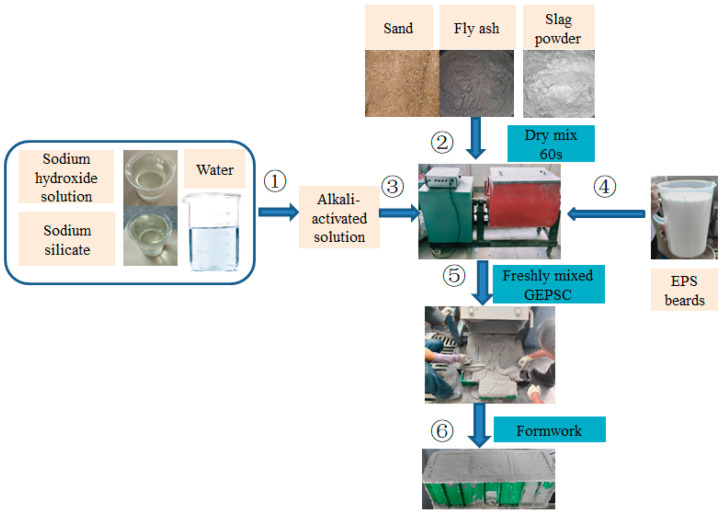
Main experimental procedure.

The alkali activator used in this study was prepared by combining sodium hydroxide solution and sodium silicate solution. Before testing, sodium hydroxide solution, sodium silicate solution, and mixing water were blended according to the designed mix proportions to obtain the required alkali-activating solution. During preparation of geopolymer EPS concrete, washed sea sand, slag powder, and fly ash were first added to a mixer and dry-mixed for 60 s to ensure preliminary uniformity. The pre-prepared alkali-activating solution was then added, and mixing was continued until a homogeneous paste formed. EPS foam particles were subsequently added, followed by the remaining mixing water, and mixing continued until the EPS particles were uniformly dispersed in the paste. The main specimen preparation procedure is shown in Figure 2.

Specimen preparation was carried out in accordance with the Standard for Test Methods of Concrete Physical and Mechanical Properties (GB/T 50081-2019) [32]. For each GEPSC mix proportion, four groups of 100 mm × 100 mm × 100 mm cube specimens, two groups of 100 mm × 100 mm × 400 mm prism specimens, and one group of 100 mm × 100 mm × 300 mm prism specimens were prepared, with each group containing three parallel specimens. After casting, the specimens were kept under natural curing conditions for 24 h, demolded, and then transferred to a standard curing room until the specified age for testing. The standard curing conditions were maintained at a temperature of 20 ± 2 °C, a relative humidity of not less than 95%, and normal atmospheric pressure.

### 2.4. Test Methods

To investigate the mechanical properties of geopolymer EPS concrete with different mix proportions, cube compression, axial compression, splitting tensile, and flexural strength tests were conducted in accordance with GB/T 50081-2019 [32]. Cube compressive strength and splitting tensile strength were tested using 100 mm × 100 mm × 100 mm cube specimens; axial compressive strength was tested using 100 mm × 100 mm × 300 mm prism specimens; and flexural strength was tested using 100 mm × 100 mm × 400 mm prism specimens. The specimens were tested after 28 d of standard curing. Three parallel specimens were tested for each mix proportion, and the arithmetic mean was taken as the final strength value. The tests were performed using an electro-hydraulic servo testing machine under displacement control at a loading rate of 3 mm/min.

## 3. Results and Discussion

### 3.1. Test Results

The dry density, cube compressive strength, axial compressive strength, splitting tensile strength, flexural strength, and elastic modulus of GEPSC obtained in this study are listed in Table 4.

### 3.2. Dry Density

**Figure 3:**
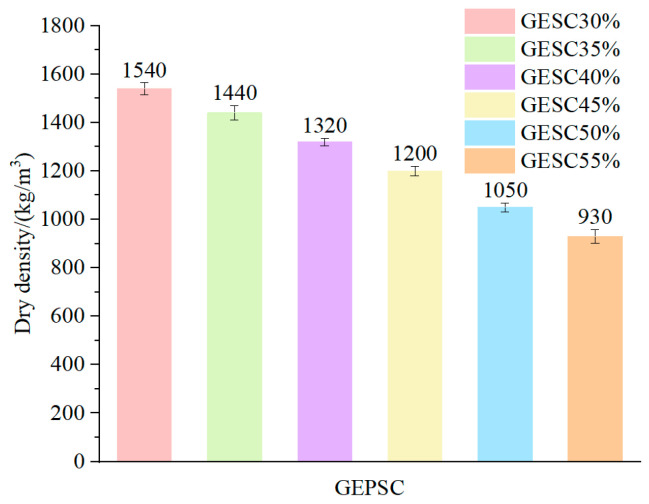
Dry density.

The dry density results of GEPSC specimens with different EPS contents are shown in Figure 3. As shown in the figure, the dry density continuously decreases with increasing EPS content, from 1540 kg/m^3^ for GEPSC30% to 930 kg/m^3^ for GEPSC55%, corresponding to a reduction of approximately 39.6%. This is because the density of EPS foam particles is much lower than that of the geopolymer matrix and fine aggregate. As the EPS volume content increases, the proportion of the low-density lightweight phase in the specimens increases, leading to a gradual decrease in overall dry density. Therefore, EPS content is an important factor for controlling the dry density of GEPSC and provides a basis for the subsequent analysis of mechanical properties.

### 3.3. Cube Compressive Strength

**Figure 4:**
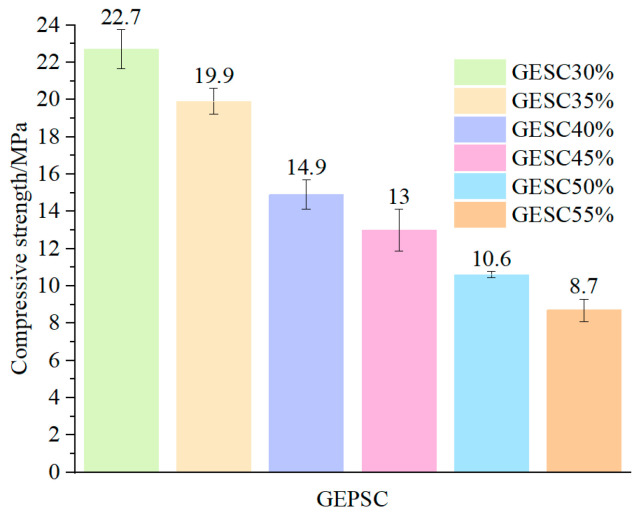
Cube compressive strength.

Figure 4 shows the cube compressive strength results of GEPSC specimens with different EPS contents. The compressive strength decreases continuously with increasing EPS content. The GEPSC30% group exhibits the highest strength, 22.7 MPa, whereas the strength decreases to the lowest level when the EPS content reaches 55%. This behavior is attributed to the fact that EPS foam particles are a lightweight phase with low strength and low stiffness. Increasing their content weakens the continuity of the geopolymer matrix and the overall load-bearing skeleton while increasing internal pores and weak interfacial zones. As a result, cracks and local damage are more likely to develop under compression. Combined with the dry density results, the decrease in compressive strength is closely related to the increased lightweighting degree of the specimens. Thus, EPS content is a key factor affecting the cube compressive strength of GEPSC, and both lightweighting and load-bearing performance should be considered in mix design.

### 3.4. Axial Compressive Strength

(1) Failure Phenomena

**Figure 5:**
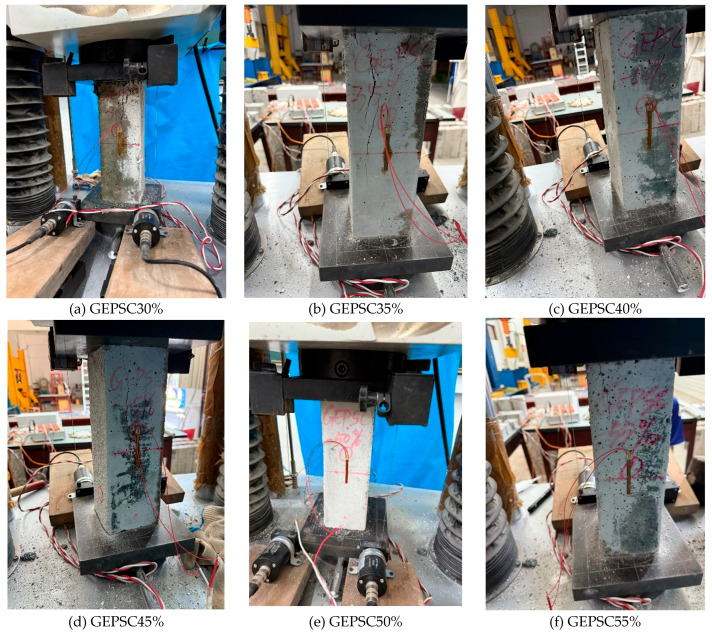
Failure modes under axial compression.

The failure modes of the axially compressed specimens are shown in Figure 5. At the initial loading stage, the specimens were in an elastic compression stage, and no obvious surface cracks were observed. As the load increased, fine vertical cracks gradually developed along the height of the specimen sides, accompanied by slight local spalling. When the load approached the peak value, cracks propagated rapidly and connected with each other, and the load-bearing capacity began to decrease.

**Figure 6:**
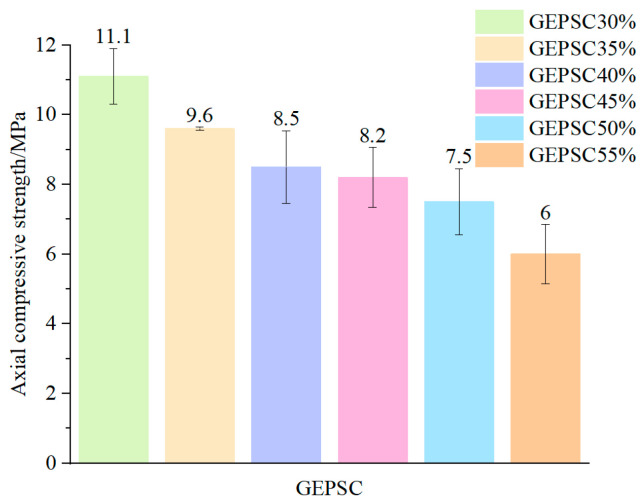
Axial compressive strength.

Figure 6 shows that the failure modes differed among specimens with different EPS contents. At low EPS contents, the matrix was relatively dense, and crack development under compression was concentrated. Wide longitudinal cracks were likely to form on the sides, accompanied by local splitting and corner spalling, indicating pronounced brittle failure. With increasing EPS content, the failure mode gradually changed from concentrated through-cracks to dispersed fine cracks. The specimen surfaces were mainly characterized by fine network cracks and local crushing and spalling, and the overall failure process became relatively gradual.

This behavior mainly results from the low strength, low elastic modulus, and relatively high deformability of EPS foam particles. Under axial loading, EPS particles can undergo a certain degree of compressive deformation, thereby relieving internal stress concentration and transforming crack propagation from a concentrated pattern to a dispersed pattern. However, increasing EPS content also weakens the continuity and integrity of the load-bearing skeleton of the geopolymer matrix, leading to a reduction in axial compressive strength. Overall, specimens with low EPS content mainly exhibit longitudinal splitting failure, whereas specimens with high EPS content show combined crushing, spalling, and dense cracking.

The above failure behavior can be further interpreted from the perspective of the microstructure of geopolymer EPS concrete. Previous SEM observations of GEPSC have shown that the interfacial characteristics between EPS particles and the geopolymer matrix are closely related to crack initiation and propagation [26]. A relatively compact geopolymer matrix and a stable matrix–EPS particle interface can help maintain the integrity of the composite and delay the rapid development of localized cracks. Therefore, under axial compression, specimens with higher EPS contents tended to exhibit more dispersed fine cracks and a more gradual failure process rather than sudden brittle splitting. Nevertheless, excessive EPS particles still reduce the continuity of the load-bearing geopolymer skeleton and increase the proportion of low-stiffness phases, which ultimately leads to a decrease in axial compressive strength.

(2) Axial Compressive Strength

The axial compressive strength of GEPSC specimens with different EPS contents is shown in Figure 6. With increasing EPS foam particle volume content, the axial compressive strength generally decreases. The GEPSC30% group has the highest axial compressive strength, 11.1 MPa, whereas in the GEPSC55% group, it decreases to 6.0 MPa, approximately 45.9% lower than that of GEPSC30%. This indicates that increasing EPS content significantly reduces the axial compressive load-bearing capacity of GEPSC.

This reduction occurs because EPS foam particles are a lightweight phase with low strength and low stiffness. When their content increases, the continuity of the geopolymer matrix is weakened, internal pores and weak interfacial zones increase, and local crushing, crack propagation, and instability of the load-bearing skeleton are more likely to occur under axial loading. Therefore, EPS content is an important factor affecting the axial compressive strength of GEPSC, and the balance between lightweighting and axial load-bearing capacity should be considered in mix design.

(3) Elastic Modulus

**Figure 7:**
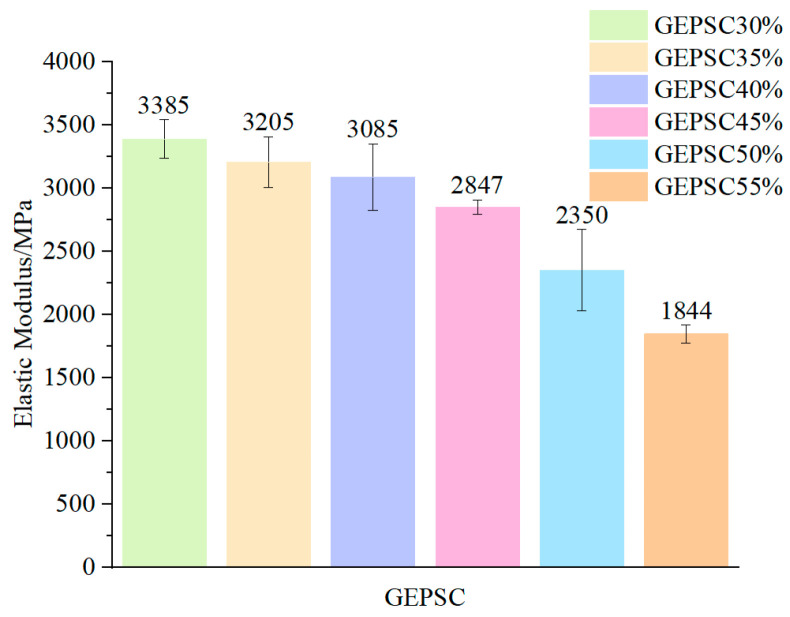
Elastic modulus.

As shown in Figure 7, the elastic modulus of GEPSC decreases overall with increasing EPS content. When the EPS content increases from 30% to 55%, the elastic modulus decreases from 3385 MPa to 1844 MPa, corresponding to a reduction of approximately 45.5%. The decrease is relatively moderate in the 30–45% content range, whereas it becomes more pronounced when the EPS content increases to 50% and 55%, indicating that high EPS contents significantly weaken the overall stiffness of GEPSC.

(4) Ratio of Axial Compressive Strength to Cube Compressive Strength

**Figure 8:**
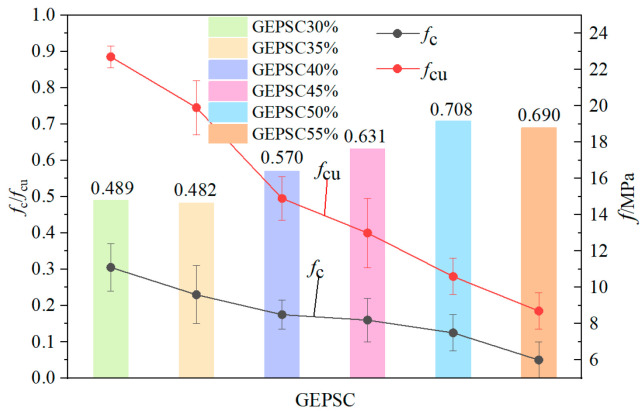
Ratio of axial compressive strength to cube compressive strength.

Based on the preceding strength results and Figure 8, both cube compressive strength and axial compressive strength decreased with increasing EPS content, indicating that EPS particles weakened the compressive load-bearing capacity of GEPSC. However, the ratio of axial compressive strength to cube compressive strength showed an overall increasing trend after a slight decrease at 35% EPS content. The ratio increased from 0.489 for GEPSC30% to 0.708 for GEPSC50% and then slightly decreased to 0.690 for GEPSC55%. This indicates that the difference between cube compressive strength and axial compressive strength gradually decreased with increasing EPS content. This phenomenon may be attributed to the reduced overall stiffness of the material at high EPS contents, which promotes more dispersed crack development and changes the failure mode from concentrated brittle failure to progressive crushing failure. As a result, the strength difference between axial compression and cube compression becomes smaller. From an engineering perspective, the axial compressive strength of GEPSC should not be estimated simply using a fixed conversion coefficient for conventional concrete. Instead, a conversion relationship considering EPS content or cube compressive strength should be established to improve the rationality of structural design parameter selection.

### 3.5. Splitting Tensile Strength

**Figure 9:**
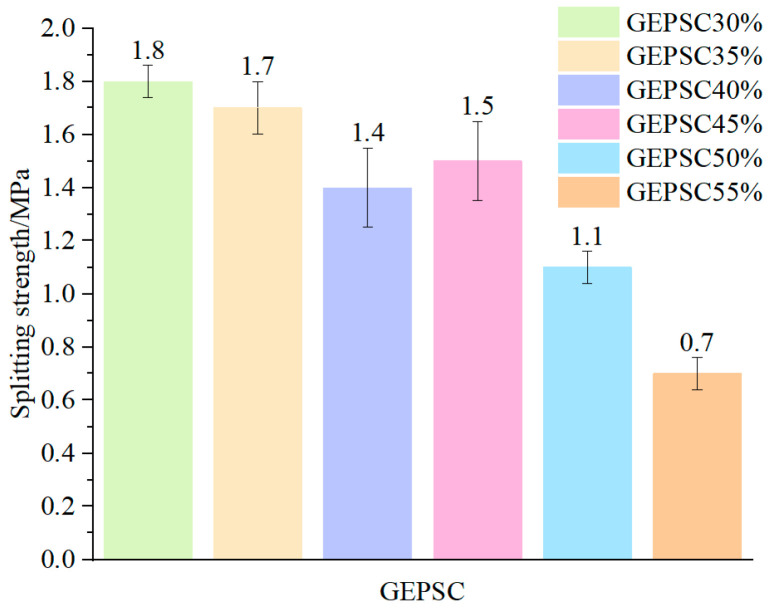
Splitting tensile strength.

Figure 9 shows the variation in splitting tensile strength of GEPSC specimens with different EPS contents. The splitting tensile strength generally decreases with increasing EPS content, from 1.8 MPa for GEPSC30% to 0.7 MPa for GEPSC55%, corresponding to a reduction of approximately 61.1%. The splitting tensile strength of the 45% group is slightly higher than that of the 40% group, but the increase is small and may be related to the uniformity of EPS particle distribution, local pore differences, interfacial bonding conditions, and test scatter. Therefore, it should not be regarded as direct evidence that increasing EPS content improves tensile strength. Overall, the low strength and low stiffness of EPS foam particles weaken the continuity of the geopolymer matrix and increase internal pores and weak interfacial zones, making cracks more likely to propagate under splitting load and causing splitting tensile strength to decrease with increasing EPS content.

### 3.6. Flexural Test Results

(1) Failure Phenomena

**Figure 10:**
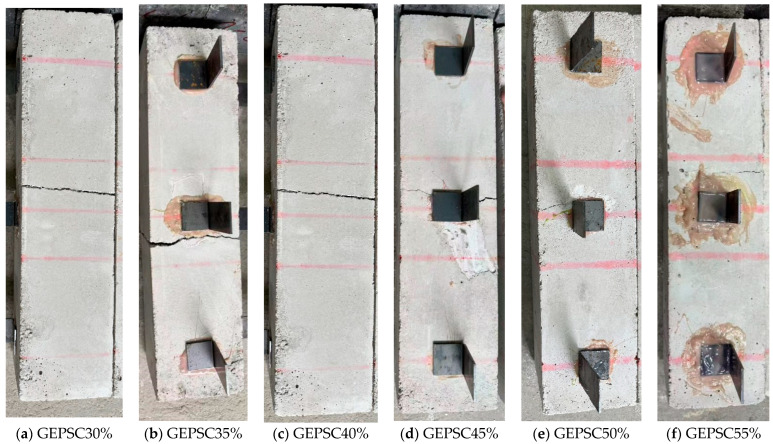
Flexural failure modes.

The failure modes of the flexural test specimens are shown in Figure 10. At the initial loading stage, the specimens worked in the elastic stage, the midspan deflection increased approximately linearly with load, and no visible cracks were observed on the specimen surface. As the load continued to increase, fine vertical cracks first appeared in the tensile zone at the bottom of the midspan. When the load approached the peak value, the main crack rapidly propagated upward, the crack width increased significantly, and the load-bearing capacity began to decrease.

The failure modes of specimens with different EPS contents show that the post-failure crack width generally decreases as the EPS foam particle content increases, and the fracture mode gradually changes from obvious brittle fracture to more ductile failure. When the EPS content is less than 45%, the specimens are usually penetrated by the main crack and fractured into two parts after failure, showing pronounced brittle behavior. When the EPS content is greater than 45%, the main crack is not fully connected after flexural failure, and the overall integrity is better maintained, indicating that increasing EPS particles improves the deformation capacity and ductility of the specimens to some extent.

Overall, although increasing EPS content reduces the flexural load-bearing capacity of the specimens, it slows the rapid penetration of cracks and changes the failure process from sudden fracture to progressive cracking, indicating better deformation compatibility.

(2) Flexural Strength

**Figure 11:**
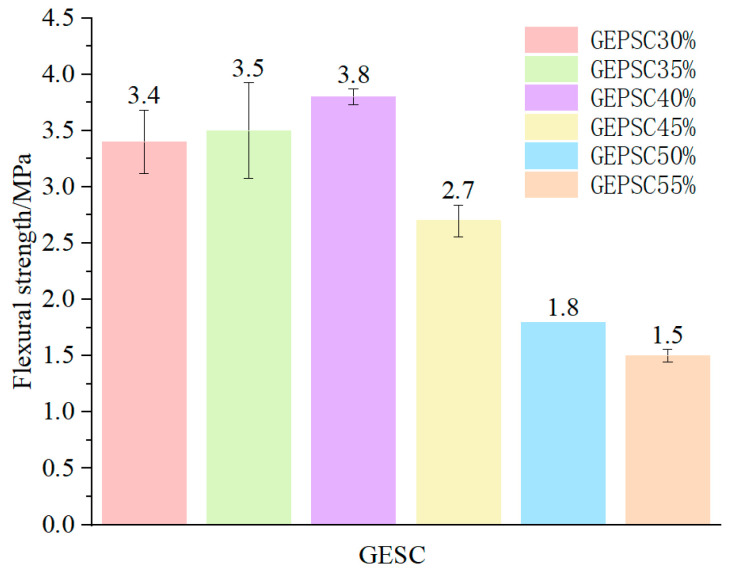
Flexural strength.

Figure 11 shows the variation in flexural strength of GEPSC specimens with different EPS foam particle volume contents. As shown in Figure 11, the flexural strength generally decreases with increasing EPS content, although some fluctuation occurs in the 30–40% content range. The flexural strength of the 40% group is slightly higher than those of the 30% and 35% groups, which may be related to the uniformity of EPS particle distribution, local pore differences, interfacial bonding conditions, and test scatter during specimen preparation. It should not be simply interpreted as an enhancement effect of increasing EPS content on flexural strength.

When the EPS content further increases to 45%, 50%, and 55%, the flexural strength decreases markedly, indicating that higher EPS contents significantly weaken the flexural performance of GEPSC. The main reason for this is that EPS foam particles have low strength and stiffness. As their volume fraction increases, the continuity of the geopolymer matrix decreases, and internal pores and weak EPS particle-paste interfacial zones increase. Under flexural tensile stress, cracks are more likely to initiate and propagate, leading to a decrease in flexural load-bearing capacity. Overall, increasing EPS content has an adverse effect on the flexural strength of GEPSC, and the slight fluctuation at low contents mainly reflects the scatter of test results for lightweight concrete specimens.

### 3.7. Analysis of Parameter Relationships

(1) Relationship between Dry Density and Axial Compressive Strength

**Figure 12:**
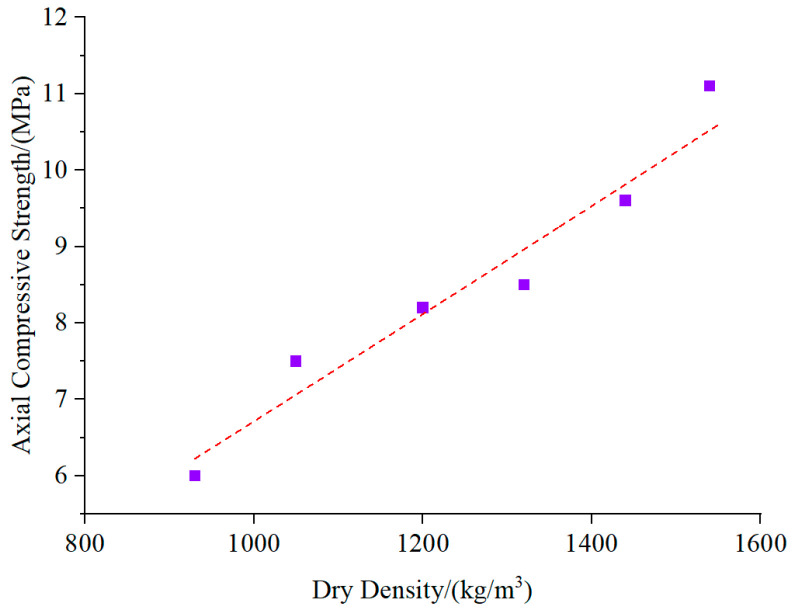
Relationship between axial compressive strength and dry density.

To reveal the internal relationship between the lightweighting degree and axial compressive performance of geopolymer EPS concrete, fitting analysis was performed on the dry density and axial compressive strength results of specimens with different EPS contents. The results are shown in Figure 12. As shown in Figure 12, the axial compressive strength of geopolymer EPS concrete generally increases with increasing dry density, indicating that dry density is an important factor affecting its axial compressive performance.

Based on the experimental data, a power function was used to fit the relationship between dry density and axial compressive strength, yielding Equation (1):

*f*_cp_ = 0.00505ρ_d_^1.04129^ (1)

where *f_cp_* is axial compressive strength (MPa), and ρ_d_ is dry density (kg/m^3^).

The fitting results show that the correlation coefficient R^2^ is 0.93478, indicating that the model can effectively describe the relationship between dry density and axial compressive strength of geopolymer EPS concrete within the experimental range. The fitted equation can be used to predict the axial compressive strength of GEPSC from dry density.

(2) Relationship between Dry Density and Cube Compressive Strength

**Figure 13:**
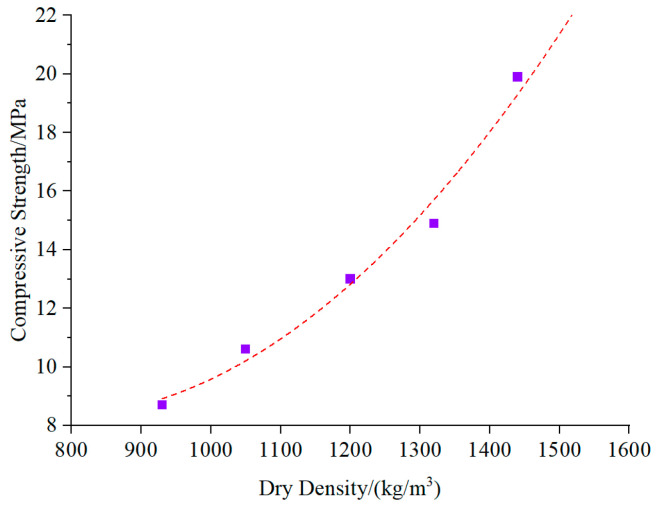
Relationship between cube compressive strength and dry density.

To further reveal the internal relationship between the lightweighting degree and compressive load-bearing capacity of geopolymer EPS concrete, fitting analysis was conducted for cube compressive strength at different dry densities. The results are shown in Figure 13. As shown in Figure 13, cube compressive strength increases significantly with dry density, indicating that dry density is an important parameter affecting the compressive performance of geopolymer EPS concrete. Based on the experimental data, a quadratic function was used to fit the relationship between dry density and cube compressive strength, yielding Equation (2):

*f*_cu_ = 7.01209 × 10^−6^ρ_d_^2^ − 6.09114 × 10^−5^ρ_d_^1.04129^ + 2.8094 (2)

where *f_cu_* is cube compressive strength (MPa), and ρ_d_ is dry density (kg/m^3^).

The fitting results show that the correlation coefficient R^2^ is 0.99511, indicating that the model effectively characterizes the relationship between dry density and cube compressive strength within the experimental range. Therefore, this model can be used to predict cube compressive strength.

(3) Relationship between Axial Compressive Strength and Cube Compressive Strength

Axial compressive strength and cube compressive strength are important indicators for evaluating the compressive performance of concrete materials. Cube compressive strength is relatively easy to test and is commonly used as a basic index for strength grading and performance evaluation, whereas axial compressive strength more closely reflects the mechanical response of members under actual compression and is a key parameter in structural design and bearing capacity analysis. Therefore, establishing a quantitative relationship between these two strength indices is important for improving the mechanical property evaluation system of geopolymer EPS concrete and enabling rational conversion between different strength parameters.

**Figure 14:**
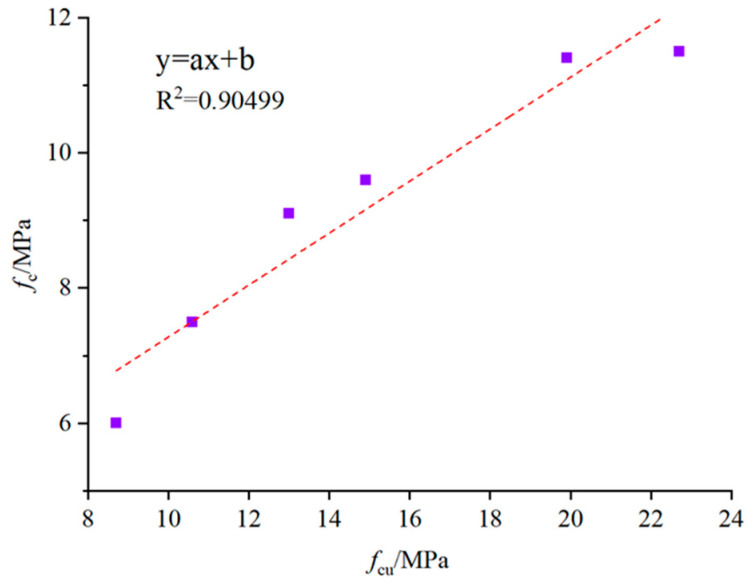
Relationship between axial compressive strength and cube compressive strength.

To reveal the internal relationship between axial compressive strength and cube compressive strength of geopolymer EPS concrete, fitting analysis was performed on the test results of specimens with different EPS contents. The results are shown in Figure 14. As shown in Figure 14, axial compressive strength increases with cube compressive strength, and the two indices show good linear correlation. Based on the experimental data, a linear function was used for fitting, yielding Equation (3):

(3) $$f_{c}=0.38459f_{cu}+3.42726$$

where *f_c_* is axial compressive strength (MPa), and *f_cu_* is cube compressive strength (MPa),

The fitting results show that the correlation coefficient R^2^ is 0.90499, indicating that the fitted equation can effectively characterize the relationship between axial compressive strength and cube compressive strength of geopolymer EPS concrete within the experimental range. This relationship makes it possible to predict axial compressive strength from cube compressive strength and provides experimental support for strength evaluation, structural design parameter selection, and mix optimization of geopolymer EPS concrete.

The test results show that the ratio of axial compressive strength to cube compressive strength ranges from 0.489 to 0.708, indicating that although the two parameters are strongly correlated, obvious differences remain under different compression boundary conditions. During cube compression, the end restraint is stronger and lateral deformation is partly restricted, resulting in a relatively higher measured strength. In contrast, axial compression specimens have a larger height and weaker lateral restraint during loading. Internal pores and weak interfacial zones between EPS particles and the geopolymer paste more readily induce longitudinal crack development, resulting in lower axial compressive strength than cube compressive strength.

Establishing the relationship between axial compressive strength and cube compressive strength helps reveal the strength conversion behavior of geopolymer EPS concrete under different compression states and provides a reference for mechanical parameter prediction and design value selection in engineering applications.

(4) Relationship between Cube Compressive Strength and Elastic Modulus

Elastic modulus is an important parameter characterizing the stiffness and deformation capacity of concrete materials, and it is also a key mechanical index for structural deformation calculation, stress analysis, and engineering design. By comparison, cube compressive strength testing is mature and provides data more conveniently. Therefore, establishing a quantitative relationship between cube compressive strength and the elastic modulus of geopolymer EPS concrete is useful for predicting material stiffness from conventional strength indices and for improving the mechanical property evaluation system.

**Figure 15:**
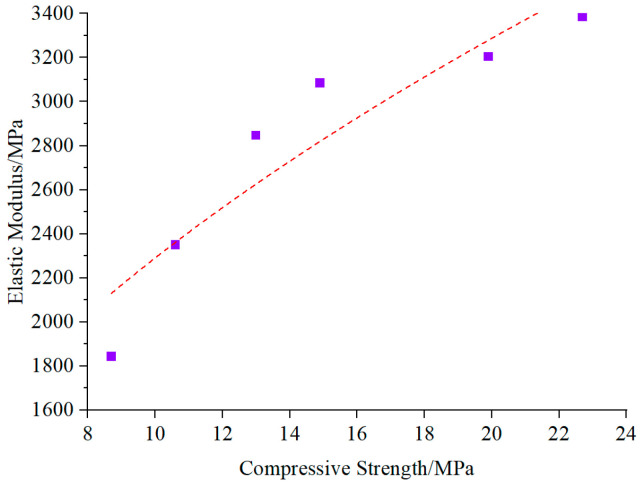
Relationship between cube compressive strength and elastic modulus.

To reveal the internal relationship between strength development and stiffness evolution of geopolymer EPS concrete, fitting analysis was conducted using the cube compressive strength and elastic modulus of specimens under standard curing conditions. The results are shown in Figure 15. As shown in Figure 15, the elastic modulus increases overall with increasing cube compressive strength, and the two parameters show good correlation. Based on the experimental data, a power function was used for fitting, yielding Equation (4):

(4) $$E_{c}=488.11473\cdot f_{cu}^{0.66312}$$

where E_c_ is elastic modulus (MPa), and *f_cu_* is cube compressive strength (MPa).

The fitting results show that the correlation coefficient R^2^ is 0.9125, indicating that the equation can effectively describe the relationship between cube compressive strength and elastic modulus of geopolymer EPS concrete within the experimental range. This relationship makes it possible to predict elastic modulus from compressive strength and provides experimental support for stiffness evaluation, deformation calculation, structural analysis, and mix optimization of geopolymer EPS concrete.

From the perspective of material structure, as cube compressive strength increases, the internal porosity of the specimens decreases, the continuity of the geopolymer matrix improves, weak interfacial zones between EPS particles and the paste are reduced, and the overall load-bearing skeleton becomes more stable. Consequently, the elastic modulus increases. Conversely, when EPS content is high, the proportion of the low-stiffness lightweight phase increases, internal pores and interfacial defects increase, and the material’s resistance to elastic deformation decreases.

Overall, establishing the relationship between cube compressive strength and elastic modulus not only helps reveal the coupled strength–stiffness evolution of geopolymer EPS concrete but also provides a reference for mechanical parameter prediction and design value selection for this type of lightweight geopolymer material in engineering applications.

(5) Model Applicability and Engineering Implications

As shown in Table 5, the mechanical parameter relationships established in this study exhibit good fitting performance, with all correlation coefficients R^2^ greater than 0.90. This indicates that the models can effectively reflect the performance variation of GEPSC within the experimental range. Among them, the relationship between dry density and cube compressive strength has the highest correlation, with R^2^ reaching 0.99511, suggesting that dry density can serve as an important control index for GEPSC mix design and preliminary strength prediction. Cube compressive strength also correlates well with axial compressive strength and elastic modulus, enabling estimation of axial compressive strength and stiffness parameters from conventional compression test results. Overall, these empirical equations can provide references for strength prediction, stiffness estimation, and mix optimization of GEPSC in lightweight wall panels, thermal insulation components, and non-load-bearing envelope structures.

### 3.8. Lightweighting Efficiency and Strength Retention Analysis

The dry density reduction ratio, strength retention ratio, and specific strength were calculated to evaluate the lightweighting efficiency and strength retention capacity of GEPSC [33,34,35,36]. The GEPSC30% group was taken as the reference group.

R_d_ = (ρ_d,30_ − ρ_d,i_)/ρ_d,30_ × 100% (5)

R_f_ = f_cu,i_/f_cu,30_ × 100% (6)

f_sp_ = f_cu,i_/(ρ_d,i_/1000) (7)

where R_d_ is the dry density reduction ratio; R_f_ is the strength retention ratio; f_sp_ is the specific strength; ρ_d,30_ and f_cu,30_ are the dry density and cube compressive strength of the GEPSC30% group, respectively; and ρ_d,i_ and f_cu,i_ are the dry density and cube compressive strength of the i-th group, respectively.

The calculated results are shown in Table 6. As shown in Table 6, as the EPS volume content increases from 30% to 55%, the dry density reduction ratio of GEPSC gradually increases to 39.61%, indicating that increasing EPS content can significantly improve the lightweighting degree of the material. However, the strength retention ratio decreases from 100.00% to 38.33%, indicating that EPS particles reduce material density while also markedly weakening the compressive load-bearing capacity of GEPSC. These trends show that the lightweighting process of GEPSC is accompanied by an obvious strength reduction, and a trade-off exists between weight reduction and strength loss.

For different EPS contents, when the EPS content increases from 30% to 35%, the dry density reduction ratio of GEPSC is 6.49%, while the strength retention ratio remains 87.67%. The specific strength decreases slightly from 14.74 MPa/(t/m^3^) to 13.82 MPa/(t/m^3^), indicating that a low EPS content has a relatively limited weakening effect on material strength and load-bearing efficiency per unit density. When the EPS content further increases to 40%, the dry density reduction ratio rises to 14.29%, but the strength retention ratio decreases to 65.64% and the specific strength decreases to 11.29 MPa/(t/m^3^), indicating more pronounced strength loss and an enhanced weakening effect of EPS particles on the continuity and load-bearing skeleton of the geopolymer matrix.

When the EPS content increases from 40% to 45%, the dry density reduction ratio of GEPSC increases from 14.29% to 22.08%, whereas the strength retention ratio decreases from 65.64% to 57.27%, and the specific strength decreases only from 11.29 MPa/(t/m^3^) to 10.83 MPa/(t/m^3^). This result indicates that in the 40–45% content range, the material can achieve a clear lightweighting effect while maintaining a relatively small decrease in compressive strength per unit density, suggesting a favorable balance between lightweighting efficiency and strength retention.

When the EPS content further increases to 50% and 55%, the dry density reduction ratios reach 31.82% and 39.61%, respectively, and the lightweighting effect is further enhanced. However, the strength retention ratios decrease to 46.70% and 38.33%, respectively, and the specific strengths decrease to 10.10 MPa/(t/m^3^) and 9.35 MPa/(t/m^3^). This indicates that although high EPS contents can significantly reduce the self-weight of GEPSC, the low strength, low stiffness, and weak interfacial characteristics of EPS further weaken the load-bearing skeleton, resulting in marked reductions in compressive strength and load-bearing efficiency per unit density.

Considering the density reduction ratio, strength retention ratio, and specific strength, a higher EPS content is not always preferable. GEPSC specimens with low EPS contents have higher strength and specific strength but limited lightweighting effects, whereas specimens with high EPS contents have clear lightweighting effects but insufficient strength retention capacity. In comparison, EPS contents of 40–45% provide a relatively favorable balance between lightweighting and strength retention. If strength retention is prioritized, 40% EPS content can be considered first; if weight reduction is emphasized while maintaining adequate compressive performance, 45% EPS content shows better overall performance. Therefore, for lightweight wall panels, thermal insulation components, and non-load-bearing envelope structures, an EPS content of 40–45% can serve as an important reference range for GEPSC mix optimization.

### 3.9. Performance Comparison Between GEPSC and EPSC and Analysis of Engineering Applicability

(1) Comparison at Similar Density Levels

To further evaluate the performance level of the geopolymer EPS concrete prepared in this study, conventional cement-based EPS concrete with similar dry density was selected as a reference material for comparison. It should be noted that GEPSC and EPSC differ in binder system and mix composition; therefore, this comparison is not a single-factor controlled experiment under strictly identical variables, and the strength differences cannot be simply attributed to differences in binder system. The comparison is mainly intended to analyze the mechanical performance level and engineering application potential of the GEPSC prepared in this study at similar lightweighting levels. As shown in Table 7, GEPSC exhibited 23.5–49.5% higher strength at the same density grade, indicating that it retains a certain compressive load-bearing capacity while maintaining low dry density. This suggests that geopolymer EPS concrete deserves further investigation for lightweight thermal insulation components, non-load-bearing envelope structures, and low-carbon building materials. However, because the mix proportion parameters of different material systems are not fully consistent, further comparative tests under the same EPS content, similar water-binder ratio, and consistent curing conditions are needed to verify the influence mechanism of the geopolymer binder system on the mechanical properties of EPS concrete.

(2) Comparison at Similar Strength Levels

As shown in Table 8, under similar cube compressive strength conditions, the mechanical properties of GEPSC differ markedly from those of cement-based EPSC. When the strength is approximately 9 MPa, the axial compressive strength of GEPSC is 6.0 MPa, lower than the 7.78 MPa of cement-based EPSC, and its elastic modulus is 1844 MPa, also lower than the 6855 MPa of EPSC. When the strength is approximately 19 MPa, the axial compressive strength and elastic modulus of GEPSC are also lower than those of the corresponding cement-based EPSC. This indicates that at similar cube compressive strength levels, GEPSC has relatively lower stiffness and axial compressive load-bearing capacity, stronger deformability, and weaker resistance to axial compressive deformation.

Regarding the ratio of axial compressive strength to cube compressive strength, the values for GEPSC55% and GEPSC35% are 0.69 and 0.48, respectively, both lower than those of cement-based EPSC with similar strength, namely 0.87 and 0.72. This indicates that the empirical conversion coefficients used for ordinary cement-based EPS concrete cannot be directly applied when converting the cube compressive strength of GEPSC to axial compressive strength. In engineering applications, if GEPSC is used in lightweight wall panels, thermal insulation components, or non-load-bearing envelope structures, its lower elastic modulus and axial compressive strength should be carefully considered. The conversion coefficient for axial compressive strength should be appropriately reduced, and deformation control and stiffness verification should be strengthened.

## 4. Conclusions

This study investigated the effect of EPS volume content on the physical and mechanical properties of GEPSC and compared its performance with cement-based EPS concrete. The main conclusions are as follows:

(1) As EPS volume content increased from 30% to 55%, dry density decreased by 39.6%, while cube compressive strength, axial compressive strength, splitting tensile strength, and elastic modulus decreased by 61.7%, 45.9%, 61.1%, and 45.5%, respectively.

(2) The 40–45% EPS content range provided the best balance between lightweighting and strength retention, with a dry density reduction ratio of 14.29–22.08% and a strength retention ratio of 57.27–65.64%. This range can serve as a reference for GEPSC mix design in lightweight envelope and thermal insulation components.

(3) The empirical relationships among the main mechanical parameters showed good fitting accuracy, with all R^2^ values exceeding 0.90. These models provide useful tools for preliminary strength prediction, stiffness estimation, strength conversion, and mix optimization of GEPSC within the tested range.

(4) Compared with cement-based EPS concrete, GEPSC showed a 23.5–49.5% higher cube compressive strength at the same density grade, indicating better strength retention. However, its lower elastic modulus and axial compressive strength at similar strength levels suggest that deformation control and axial-to-cube strength conversion should be considered in engineering applications.

## Figures and Tables

**Table 1:** Chemical composition and physical parameters of water glass [26].

Raw Material	SiO_2_/%	Na_2_O/%	Density (kg/m^3^)	Baume Degree (Bé)	Modulus M	Transparency
Water glass	27.5–30.5	8.5–10.5	1394–1408	41–42	3.32	≥85

**Table 2:** Chemical composition and physical parameters of fly ash and slag powder [26].

Chemical Composition (%)	Al_2_O_3_	SiO_2_	CaO	MgO	Fe_2_O_3_	Na_2_O	K_2_O	SO_3_	TiO_2_
Fly ash	42.35	34.48	4.62	0.60	9.88	0.48	1.42	2.15	2.51
Slag powder	15.7	30.8	41.5	7.7	0.3	0.7	/	1.1	/

**Table 3:** Mix proportions of GEPSC.

Mix ID	Fly Ash (kg/m^3^)	Slag Powder (kg/m^3^)	Sand (kg/m^3^)	EPS (L/m^3^)	Sodium Hydroxide (kg/m^3^)	Sodium Silicate (kg/m^3^)	Water (kg/m^3^)	Superplasticizer (kg/m^3^)
GEPSC30%	110	440	800	480	44	185	260.5	7
GEPSC35%	110	440	700	570	44	185	260.5	7
GEPSC40%	110	440	600	670	44	185	260.5	7
GEPSC45%	110	440	500	770	44	185	260.5	7
GEPSC50%	110	440	400	880	44	185	260.5	7
GEPSC55%	110	440	300	1000	44	185	260.5	7

**Table 4:** Mechanical property test results of GEPSC.

Specimen ID	Dry Density (kg/m^3^)	Cube Compressive Strength (MPa)	Axial Compressive Strength (MPa)	Splitting Tensile Strength (MPa)	Flexural Strength (MPa)	Elastic Modulus (MPa)
GEPSC30%	1540	22.7	11.1	1.8	3.4	3385
GEPSC35%	1440	19.9	9.6	1.7	3.5	3205
GEPSC40%	1320	14.9	8.5	1.4	3.8	3085
GEPSC45%	1200	13.0	8.2	1.5	2.7	2847
GEPSC50%	1050	10.6	7.5	1.1	1.8	2350
GEPSC55%	930	8.7	6.0	0.7	1.5	1844

**Table 5:** Fitted relationships among main mechanical parameters of GEPSC and engineering applications.

Predictive Relationship	Fitting Type	Equation	Correlation Coefficient	Engineering Application
ρ_d_ → *f*_c_	Power function	*f*_cp_ = 0.00505ρ_d_^1.04129^	0.93478	Axial compressive strength prediction
ρ_d_ → *f*_cu_	Quadratic function	*f*_cu_ = 7.01209 × 10^−6^ρ_d_^2^ − 6.09114 × 10^−5^ρ_d_^1.04129^ + 2.8094	0.99511	Mix design
*f*_cu_ → *f*_c_	Linear function	$f_{c}=0.38459f_{cu}+3.42726$	0.90499	Strength conversion
*f*_cu_ → E_c_	Power function	$E_{c}=488.11473\cdot f_{cu}^{0.66312}$	0.91250	Stiffness estimation

Note: ρ_d_ is dry density, *f_cu_* is cube compressive strength, *f_c_* is axial compressive strength, and E_c_ is elastic modulus.

**Table 6:** Dry density reduction ratio, strength retention ratio, and specific strength of GEPSC.

Index	GEPSC30%	GEPSC35%	GEPSC40%	GEPSC45%	GEPSC50%	GEPSC55%
Dry density reduction ratio (%)	0.00	6.49	14.29	22.08	31.82	39.61
Strength retention ratio (%)	100.00	87.67	65.64	57.27	46.70	38.33
Specific strength [MPa/(t/m^3^)]	14.74	13.82	11.29	10.83	10.10	9.35

**Table 7:** Comparison between GEPSC and EPSC at similar density levels.

Reference or Specimen ID	Dry Density Range (kg/m^3^)	Binder System	Dry Density (kg/m^3^)	Compressive Strength (MPa)
GEPSC50%	1000–1100	Geopolymer	1050	10.6
Reference [37]	1000–1100	Cement-based	1058	7.1
References [38,39]	1000–1100	Cement-based	1065	3.4
Reference [40]	1000–1100	Cement-based	1070	6.91
Reference [41]	1200–1300	Cement-based	1258	10.53
GEPSC45%	1200–1300	Geopolymer	1200	13.0

**Table 8:** Comparison between GEPSC and EPSC at similar strength levels.

Reference or Specimen ID	Strength (MPa)	Binder System	Elastic Modulus (MPa)	Axial Compressive Strength (MPa)	*f*_c_/*f*_cu_
Reference [42]	8.97	Cement-based	6855	7.78	0.87
GEPSC55%	8.7	Geopolymer	1844	6.0	0.69
References [43,44]	19.15	Cement-based	9190	13.70	0.72
GEPSC35%	19.9	Geopolymer	3205	9.6	0.48

## Data Availability

The original contributions presented in this study are included in the article. Further inquiries can be directed to the corresponding author.
